# Enhancement of Terahertz Radiation by Surface Plasmons Based on CdTe Thin Films

**DOI:** 10.3390/nano12020290

**Published:** 2022-01-17

**Authors:** Huiyan Kong, Luyi Huang, Min Li, Ling Zhang, Heping Zeng

**Affiliations:** 1 School of Optical-Electrical and Computer Engineering, University of Shanghai for Science and Technology, Shanghai 200093, China; 192380339@st.usst.edu.cn (H.K.); minli_1220@163.com (M.L.); 2 CAS Key Laboratory of Nanophotonic Materials and Devices & Key Laboratory of Nanodevices and Applications, i-Lab, Suzhou Institute of Nano-Tech and Nano-Bionics (SINANO), Chinese Academy of Sciences, Suzhou 215123, China; luhuang2021@sinano.ac.cn; 3 State Key Laboratory of Precision Spectroscopy, East China Normal University, Shanghai 200062, China; hpzeng@phy.ecnu.edu.cn

**Keywords:** THz emission time-domain spectroscopy, cadmium telluride, metals, THz enhancement, surface plasmons

## Abstract

Terahertz (THz) time-domain spectroscopy (TDS) is a powerful tool used to characterize the surface/interface of materials, and semiconductor/metal interfaces can generate THz emission through ultrafast optical excitation, which can be further improved through the optical excitation of surface plasmons. Here, we assembled cadmium telluride (CdTe) on an AuAg alloy (Au_25_Ag_75_, wt.%) substrate and obtained five times stronger THz emission compared with silicon substrate, and found that the enhancement can be tuned by controlling the thickness of the semiconductor materials and plasmonic metal substrates. We believe that our results not only promote the development of THz emission enhancement, but also provide a straightforward way of producing small, thin, and more efficient terahertz photonic devices.

## 1. Introduction

Terahertz (THz) technology possesses the unique properties of low energy, a lack of damage, and the unique fingerprint characteristics of non-polar molecules. It has blossomed rapidly through the development of ultrafast optical technologies and hybrid materials, and has been applied in various fields [1,2,3,4,5,6], such as biological imaging, medical diagnosis, customs security, and high-speed communication. Therefore, there is an increasing demand for high-efficiency THz emission sources and high-sensitivity THz wave detectors. Semiconductor materials, such as layered centrosymmetric 2H-MoS_2_ (TMDs), InAs, graphene, and Ge crystals, feature high carrier mobility, wide band range, high current on/off and other photoelectric properties, and have been used as sources of electromagnetic radiation in the THz range [7,8,9,10,11]. Additionally, THz emission strength and efficiency can be further improved by constructing semiconductor/metal Schottky interfaces. Ramakrishnan et al. prepared a Cu_2_O/Au structure to increase THz emission and pointed out that excitation of plasmons at the interface between semiconductor and metal increased light concentration and absorption [12]. Bahk et al. reported that an emitted THz wave of graphene deposited on a gold substrate was significantly enhanced by localized surface plasmons, and they doped out a terahertz emission mechanism that related to surface-plasmon-enhanced optical rectification [13]. Huang et al. demonstrated that a surface plasmon from nano-porous gold could promote photocurrent density in cadmium telluride (CdTe) to further enhance THz emission [14].

In this study, we constructed a semiconductor/metal by depositing thin CdTe films on gold and Au_25_Ag_75_ alloy substrates with rough surfaces, and achieved nearly five times the enhancement of terahertz radiation compared with CdTe on silicon. We argue that both the thickness of CdTe and the components of metal substrates affect THz emission efficiency.

## 2. Materials and Methods

**Figure 1:**
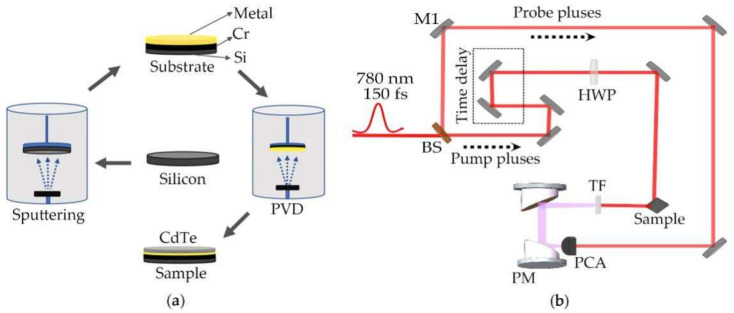
(**a**) Process of sample preparation. PVD: Physical vapor deposition; (**b**) schematic of a THz emission spectroscopy system. BS: beam splitter; M1: mirror; PM: paraboloidal mirror; HWP: half-wave plate; PCA: Photoconductive antenna; TF: Teflon wafer.

Thin CdTe [15] films were deposited on silicon (Si, 100), gold (Au), and AuAg alloy (Au_25_Ag_75_, wt.%) substrates through thermal evaporation deposition at room temperature [16]. Au and AuAg substrates were obtained by using a magnetron sputtering instrument on silicon wafers with a chromium layer for buffering at room temperature. All the metal targets were provided by Zhongnuo New Materials (Beijing, China) Technology Company. CdTe powder (Leshan Kai Yada Photoelectric Technology Co. Ltd, Leshan, Sichuan, China; 99.999% purity) was used as the evaporator source evaporated from a tungsten-wire-heated quartz boat. The thickness of the thin CdTe films was dominated by a quartz crystal monitor (FTM106) with a deposition rate of about 2 Å/s. During the process of evaporation, the vacuum pressure of the chamber was lower than 10^−4^ Pa, and the substrates kept rotating at 10 rad/s to improve the uniformity of the CdTe films. CdTe films with thicknesses of ~5, ~10, and ~15 nm were obtained by controlling evaporating time [14]. The progress of preparing samples is shown in Figure 1a.

Home-made THz time-domain spectroscopy (TDS) was used for sample measurement and a schematic of the system is shown in Figure 1b. This system used an ultrafast laser with 780 nm, 150 femtosecond pulse duration and 80 MHz repetition rate. An average pump power of 80 mW was used for irradiating samples. During the measurement, laser pulse first traveled through a beam splitter and split into two parts that were used as the pump beam with the majority of the pulse energy and the probe beam with weak energy; subsequently, the pump beam passed through a time delay system that consisted of a pair of mirrors and an optical precision moving platform to change the relative time delay between the pump beam and the probe beam. The pump beam was focalized into a spot area of ~50 μm^2^ and then irradiated the surface of samples in a 45° angle direction. The remaining pump beam was prevented by a Teflon wafer, and the generated THz pulse was focused by a pair of parabolic mirrors. Finally, the THz electromagnetic wave was collected onto a photoconductive antenna (PCA, Jena, Germany; BATOP GmbH), which was excited by the probe beam, and was transmitted into the phase-locked amplifier (Stanford SR830, Stanford, SRS) and shown by the domain-time waveforms of THz radiation.

## 3. Results and Discussions

**Figure 2:**
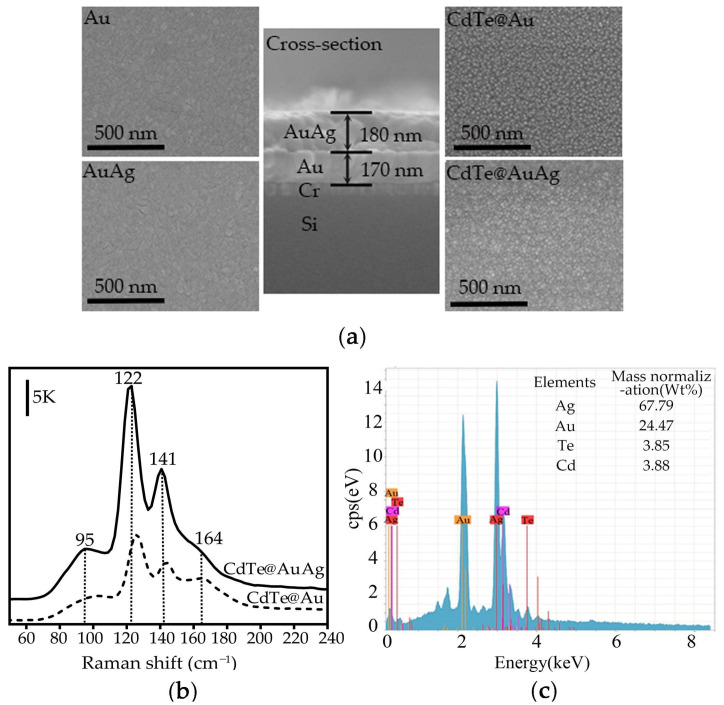
(**a**) SEM images of Au and AuAg films without and with CdTe covering, and the cross-section of the metal substrate; (**b**) Raman spectra of CdTe on Au and AuAg (the excitation wavelength of the laser is 532 nm with power set at 1 mW); (**c**) EDS spectrum of CdTe@AuAg and the element percentages of Cd and Te are shown in the top-right corner.

The surface morphologies of the Au and AuAg films without and with CdTe covering were characterized with a scanning electronic microscope (SEM, Hitachi, Tokyo, Japan) and shown in Figure 2a. It was found that the surfaces of Au and AuAg film were uniform, with similar roughness, and the thicknesses of the Au and AuAg layers were around 170 and 180 nm, respectively. After CdTe evaporation, nanoparticles were observed on the surface of the substrates. The Raman spectra of CdTe on Au and AuAg substrates were of the same shape, but the intensity collected from CdTe@Au was less than half of that from CdTe@AuAg. The characteristic Raman peak at 95 cm^−1^ indicated the low-intensity mode of CdTe [17] and the peak of 164 cm^−1^ corresponded to the LO mode from CdTe vibration [18,19,20]. The 141 cm^−1^ peak resulted in a combination of TO and E phonon modes of Cd, and the peak at 122 cm^−1^ was related to the A_1_ mode of Te in CdTe. Therefore, the main component of the deposited film was CdTe.

Energy-dispersive X-ray spectroscopy (EDS, Tokyo, Hitachi) was adopted to verify the composition of the sample [21]. As shown in Figure 2c, the energy levels of the Cd and Te elements were detected, and the atomic percentages of Cd and Te were ~3.88 and ~3.85%, respectively, which further confirmed that the main component of the evaporated films is CdTe.

**Figure 3:**
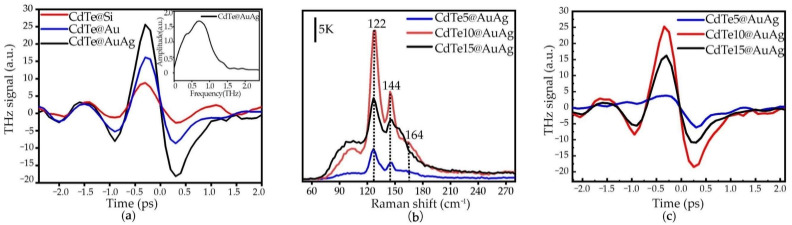
(**a**) THz emission waveforms of ~10 nm CdTe film on the Si, Au, and AuAg substrates in time-domain, and the inset is the corresponding frequency spectrum of CdTe@AuAg film; (**b**) Raman spectra of CdTe5@AuAg (the thickness of CdTe is 5 nm), CdTe10@AuAg (the thickness of CdTe is 10 nm), CdTe15@AuAg (the thickness of CdTe is 15 nm); (**c**) THz emission waveforms from CdTe5@AuAg, CdTe10@AuAg and CdTe15@AuAg films.

When the laser light beam impinged on the CdTe films, the optical absorption led to the photo-excited electron-hole pairs transiting from the valence band to the conduction band to serve as the photocarrier. This carrier, accelerated by intrinsic electric fields, generates THz wave emission [22]. It can be seen from Figure 3a that the THz radiation signals of thin CdTe film on different substrates were different. By comparing the THz signals of CdTe on the Si, Au, and AuAg alloy substrates, we found that the THz radiation amplitudes of CdTe on the AuAg and Au films were enhanced by ~5 times and ~2.5 times compared to that on the Si. For an analogous comparison in the frequency domain, the corresponding Fourier-transformed spectrum (shown in Figure 3a, inset) featured a bandwidth of about 1.6 THz. The results indicated that noble metal can enhance the THz radiation of CdTe because the electrons in the metal were excited by the laser beam, and then accumulated to form surface plasmon polaritons (SPPs) [23]. A strong localized surface electromagnetic field was generated, boosting the absorption of laser light. Due to the influence of the localized electromagnetic field, the internal electron mobility and the charge density in CdTe were adjusted again to further increase the surface depletion electric field, similar to the photo-Dember effect. With photoexcited carriers to the conduction band, both the localized electromagnetic field at the CdTe and metal interface and the intrinsic surface electric field from CdTe drove the charge transport to improve the efficiency of THz emission on the surface/interface of the material [12,24]. Meanwhile, the THz amplitude signal of CdTe@AuAg was ~2 times stronger than that of CdTe@Au. It was shown that binary alloy exhibits greater enhancement than pure metal. Compared with Au, Ag features higher electrical and thermal conductivities at room temperature and exhibits high-density photo-generated electrons. However, Au is more stable and can integrate with CdTe better due to its lower Schottky barrier [21,25,26], which results in higher carrier transmission and power conversion efficiency. Thus, AuAg alloy, which combines the best of both metals, is a good candidate for the plasmonic substrate.

The Raman spectra (RAMANtouch, Nanophoton, Osaka, Japan) of the CdTe films on AuAg with thicknesses of ~5 nm (CdTe5@AuAg), ~10 nm (CdTe10@AuAg), and ~15 nm (CdTe15@AuAg) were observed and shown in Figure 3b. By comparing the relative intensity of each peak, it can be seen that the intensity of the 122 and 144 cm^−1^ Raman peaks from CdTe10@AuAg was significantly enhanced, and was about five times larger than that from CdTe5@AuAg and 1.5 times stronger than that from CdTe15@AuAg. The corresponding THz emission waveforms of CdTe on AuAg with different thicknesses are shown in Figure 3c, and the strongest THz emission signal was also obtained from CdTe10@AuAg, which was ~5 and ~3 times stronger than that of the ~5 and ~15 nm samples, respectively. It was interesting to find that the THz emission intensity is related to the Raman intensity from CdTe.

**Figure 4:**
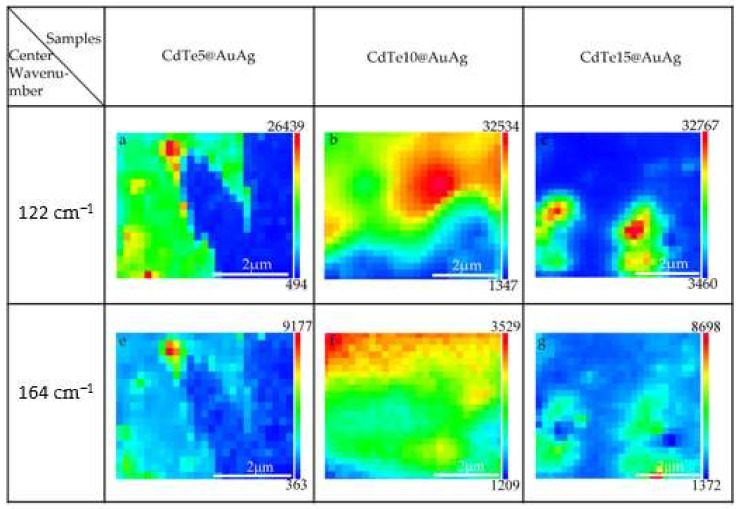
Raman mapping images of CdTe5@AuAg, CdTe10@AuAg, and CdTe15@AuAg films with characteristic peaks at 122 cm^−1^ and 164 cm^−1^.

Since both the quantity of evaporated CdTe and the surface plasmon enhancement contributed to the Raman intensity [25,27], Raman mapping images of ~5, ~10, and ~15 nm thick CdTe on the AuAg alloy films were collected and displayed in Figure 4 for further analysis, upon which 122 and 164 cm^−1^ were selected as the center wavenumbers. We observed that CdTe of ~10 nm thickness was more evenly coated on the substrate and the average strength was stronger. At the beginning of the deposition process, randomly connected CdTe networks formed because of disordered nanometer-sized CdTe clusters [21,28], as shown in Figure 2a. With the coalescence and filling of CdTe, more homogeneous films were formed, showing an increased peak intensity of 122 and 164 cm^−1^ (Figure 3b). However, with the thickness further increased to ~15 nm in Figure 3b, the peak intensity, conversely, decreased. Although thicker CdTe can effect better Raman signals, weakened SPP enhancement leads to totally decreased Raman intensity. Thus, it can be concluded that SPPs enhancement plays a key role in Raman scattering, where SPPs can generate the intense electric field to improve the optical signal of nearby materials [27]. Furthermore, SPPs are equally important for the enhancement of THz radiation; a similar variation trend can be observed in THz spectroscopy (Figure 3c). Thus, both the plasmonic enhancement and quality of thin CdTe film affect THz radiation capacity.

**Figure 5:**
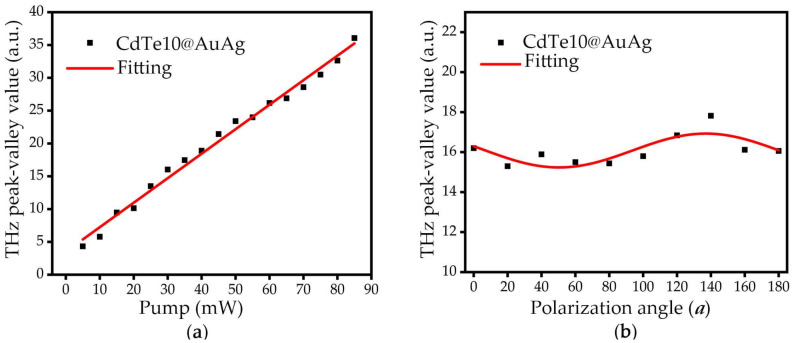
(**a**) THz peak–valley value from CdTe10@AuAg with respect to pump fluence; (**b**) tuning the pump polarization, the THz emission signal from peak to valley value in time-domain (the pump fluence used was 30 mW).

The polarity, amplitude, and phase information of the generated THz wave reflected the linear and nonlinear optical properties, which can be used to analyze the mechanism of THz radiation and enhancement. The thin CdTe film with a thickness of ~10 nm on the AuAg alloy was used for further THz wave measurement. When 780 nm laser pulses [27] illuminate the CdTe10@AuAg surface at an oblique incidence at 45°, ultrafast photocurrents are generated. These photocurrents consist of nonlinear currents ($J_{nl}$), drift currents ($J_{dri}$) and diffusion currents ($J_{dif}$), which can be distinguished via THz emission signal. The surface depletion field (E) is dominant to generate $J_{dri}$, which can be described as $J_{dri}=Ne\mu E\propto P_{photon}$, where *N* is the density of the photo-generated carrier, *e* is the electron charge, and μ is the mobility. The E is irrelevant to pump power ($P_{photon})$, while the density of the photogenerated carrier *N* is proportional to $P_{photon}.$ In addition, $J_{dif}$ is caused by the different mobility of the electrons and holes (the photo-Dember effect), and the currents can be described as $J_{dif}=Ne\mu E_{d}\propto P_{photon}^{2}$, where $E_{d}$ is the field generated by the diffusion currents. According to the formula, the diffusion currents are proportional to the pump power squared, which is nonlinear [24,29]. Referring to our experimental results, shown in Figure 5a, the peak–valley value of the THz signal exhibited a linear relation with increasing the pump power. We can infer that it was the surface depletion field, not the diffusion currents, that made the main contribution to the photocurrent.

Since the photo-generated nonlinear currents were more easily influenced by the polarization states of the ultrafast excitation pump beam, the variation of THz intensity with the polarization of the pump was used to analyze the contribution of the nonlinear effect to THz radiation [10]. The corresponding experimental results are shown in Figure 5b. A trigonometric relationship can be observed between the photocurrent and the polarization state of the laser pump. The results from the experiment reflect the non-linear property of CdTe. According to the contribution of THz amplitude from the transient photocurrent and the current of nonlinear effect, as $E_{THz}=C_{current}+C_{EFIOR}\cos2a$ [14], we can simply calculate that the contribution to THz emission of transient photocurrent was about 18 times larger than that of the nonlinear effect. In this paper, when SPPs were excited to enhance THz emission, CdTe preserved its nature.

## 4. Conclusions

We reported a THz emission enhancement of CdTe on pure metal and metal alloy thin films. The excited surface plasmon on Au and AuAg films was seen to particularly enhance THz emission by increasing the optical carrier density of CdTe. By combing with Raman scattering, we found that the thickness of CdTe exerted a slight effect on terahertz radiation and SPP enhancement mainly accounted for THz radiation enhancement. Our work suggests that it is possible to obtain stronger THz emissions from thin semiconductor films by providing a plasmonic basement and that the enhancement of the THz wave can be tuned by the components and surface morphology of metallic substrates.

## Data Availability

All data concerning this study are contained in the present manuscript or in previous articles, whose references have been provided.
